# Predicting cancer immunotherapy response from gut microbiomes using machine learning models

**DOI:** 10.18632/oncotarget.28252

**Published:** 2022-07-19

**Authors:** Hai Liang, Jay-Hyun Jo, Zhiwei Zhang, Margaret A. MacGibeny, Jungmin Han, Diana M. Proctor, Monica E. Taylor, You Che, Paul Juneau, Andrea B. Apolo, John A. McCulloch, Diwakar Davar, Hassane M. Zarour, Amiran K. Dzutsev, Isaac Brownell, Giorgio Trinchieri, James L. Gulley, Heidi H. Kong

**Affiliations:** ^1^Dermatology Branch, National Institute of Arthritis and Musculoskeletal and Skin Diseases, National Institutes of Health, Bethesda, MD 20892, USA; ^2^Biostatistics Branch, Division of Cancer Treatment and Diagnostics, National Cancer Institute, NIH, Bethesda, MD 20892, USA; ^3^Department of Medical Education, West Virginia University, Morgantown, WV 26506, USA; ^4^Translational and Functional Genomics Branch, National Human Genome Research Institute, NIH, Bethesda, MD 20892, USA; ^5^NIH Library, Division of Library Services, Office of Research Services, NIH, Bethesda, MD 20892, USA; ^6^Zimmerman Associates Inc., Fairfax, VA 22030, USA; ^7^Genitourinary Malignancies Branch, Center for Cancer Research, NCI, NIH, Bethesda, MD 20892, USA; ^8^Genetics and Microbiome Core, Laboratory of Integrative Cancer Immunology, Center for Cancer Research, NCI, NIH, Bethesda, MD 20892, USA; ^9^Department of Medicine and UPMC Hillman Cancer Center University of Pittsburgh, Pittsburgh, PA 15213, USA; ^10^Laboratory of Integrative Cancer Immunology, Center for Cancer Research, NCI, NIH, Bethesda, MD 20892, USA; ^11^Center for Immuno-Oncology, Center for Cancer Research, NCI, NIH, Bethesda, MD 20892, USA

**Keywords:** gut microbiome, immunotherapy, 16S rRNA, machine learning, metagenomics

## Abstract

Cancer immunotherapy has significantly improved patient survival. Yet, half of patients do not respond to immunotherapy. Gut microbiomes have been linked to clinical responsiveness of melanoma patients on immunotherapies; however, different taxa have been associated with response status with implicated taxa inconsistent between studies. We used a tumor-agnostic approach to find common gut microbiome features of response among immunotherapy patients with different advanced stage cancers. A combined meta-analysis of 16S rRNA gene sequencing data from our mixed tumor cohort and three published immunotherapy gut microbiome datasets from different melanoma patient cohorts found certain gut bacterial taxa correlated with immunotherapy response status regardless of tumor type. Using multivariate *selbal* analysis, we identified two separate groups of bacterial genera associated with responders versus non-responders. Statistical models of gut microbiome community features showed robust prediction accuracy of immunotherapy response in amplicon sequencing datasets and in cross-sequencing platform validation with shotgun metagenomic datasets. Results suggest baseline gut microbiome features may be predictive of clinical outcomes in oncology patients on immunotherapies, and some of these features may be generalizable across different tumor types, patient cohorts, and sequencing platforms. Findings demonstrate how machine learning models can reveal microbiome-immunotherapy interactions that may ultimately improve cancer patient outcomes.

## INTRODUCTION

In the last decade, the use of cancer immunotherapy targeting immune checkpoint inhibitors (ICIs) to boost T cell mediated cancer cell clearance has significantly improved cancer patient survival [1]. Commonly used ICIs include monoclonal antibodies targeting programmed cell death protein (PD-1) and its ligand (PD-L1) or CTL antigen 4 protein (CTLA-4) [2]. ICI combination therapy is also widely used to increase the effectiveness of other cancer treatments [3]. Although immunotherapy can significantly improve treatment outcomes amongst different cancer types as compared to other treatment modalities [4], approximately half of patients do not respond to immunotherapy [3, 5, 6]. To optimize treatment outcomes, current efforts are aimed at elucidating the internal or external features of patients or tumors that correlate with immunotherapy responsiveness [7, 8].

Increasing evidence has emerged that gut microbial communities help shape the host immune system [9–11]. Several studies have suggested specific gut bacteria can influence immunotherapy outcomes by modulating the immune responses of patients with metastatic melanoma, non–small cell lung cancer, and renal cell cancer [12–33]. Treatment responders generally exhibit increased gut microbial community diversity and are enriched in certain bacterial taxa including *Akkermansia* and *Bifidobacterium* [16, 19]. Prior studies have primarily focused on the impact of individual bacterial taxa on immunotherapy outcomes in melanoma patients. It is currently unclear whether the identified response signals are generalizable across tumor types. Furthermore, findings may not be reproducible across different patient cohorts due to geographical variation in the microbiome [34] as well as differences in sequencing platform or analysis methodology. Here, we took a tumor-agnostic approach to identify microbiome features associated with immunotherapy response from a discovery cohort of patients with nine different advanced stage cancers. To uncover common immunotherapy response signals regardless of tumor type, we conducted a combined meta-analysis integrating the discovery cohort data with three previously published 16S rRNA gene sequencing datasets from melanoma patients. Using the combined dataset, we trained and validated models with machine learning algorithms to predict patients’ clinical responses, followed by cross-sequencing-platform validation using shotgun metagenomic sequencing data.

## RESULTS

### Specific bacterial taxa associated with immunotherapy response in advanced stage cancer patients

We sought to investigate the association between patients’ baseline gut microbiomes and clinical outcomes of immunotherapy. The discovery cohort consisted of 16 patients with late-stage solid tumors enrolled in different immunotherapy trials at the National Cancer Institute (NCI). Immunotherapy clinical response rate in the cohort was 38% (6 responders and 10 non-responders) (Supplementary Table 1). Despite a male predominance in the cohort (12 of 16 male patients), patient demographics did not differ significantly between responders (83% male; mean age 58.5 ± 7.8 years) and non-responders (70% male; mean age 57.1 ± 15.5 years) (two-tailed Student’s *t*-test, *p* = 0.8140). However, antibiotic use 30 days prior to immunotherapy was more common among non-responders than responders (Fisher’s exact test, *p* = 0.2335); although not statistically significant due to a small sample size, our data align with prior observations of a negative association between recent antibiotic use and immunotherapy outcomes [35, 36].

**Figure 1:**
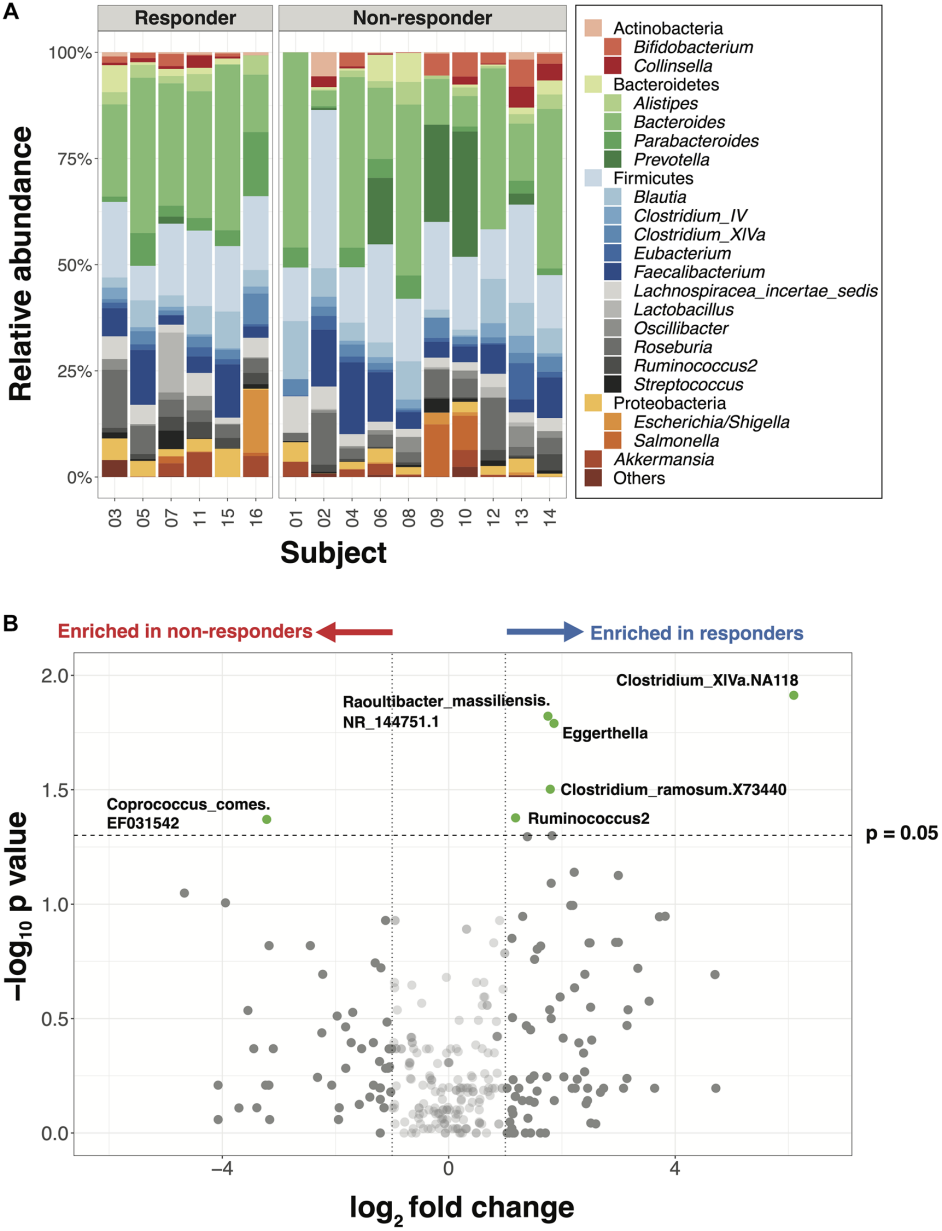
Major gut bacterial taxa of responders and non-responders from the NCI cohort. (**A**) Bar plot of phylogenetic composition of the top 25 bacterial taxa at the genus level, grouped by the response types (*n* = 16). (**B**) Volcano plot with all taxa signals from species to phylum levels. Taxa signals with greater than 2-fold change and statistically significant differences between responders and non-responders are highlighted in green (Wilcoxon rank-sum test, *p* < 0.05, unadjusted).

Sequencing data from 16 patient stool samples collected prior to immunotherapy encompassed 1583 amplicon sequencing variants (ASVs, single DNA sequences). Taxonomic profiles at the genus level showed high relative abundances of *Bacteroides* and firmicutes across all samples (Figure 1A, Supplementary Tables 2–4). Neither alpha nor beta diversity, calculated at the ASV level, differed between responder and non-responder patients.

Ten taxa, including 5 within the family Lachnospiraceae and 4 within Coriobacteriaceae, exhibited significant differences in the mean relative abundance between responders versus non-responders (Figure 1B, *p* < 0.05, unadjusted, Supplementary Table 5). Consistent with previous studies, 6 genera -- including *Akkermansia*, *Parabacteroides* and *Prevotella,* which have been reported as differentially abundant between responders and non-responders, were also found to differ in this cohort though this difference was not statistically significant (Supplementary Figure 1). To examine relationships among individual genera within the responders vs. non-responders, we performed a network analysis which highlighted that bacterial taxa are deeply interconnected in the responder network but less connected in the non-responder network (Supplementary Figure 2), suggesting a need to examine gut immunotherapy microbial predictors at a more global level.

### Gut microbial compositions distinguish responders from non-responders in the combined dataset

**Figure 2:**
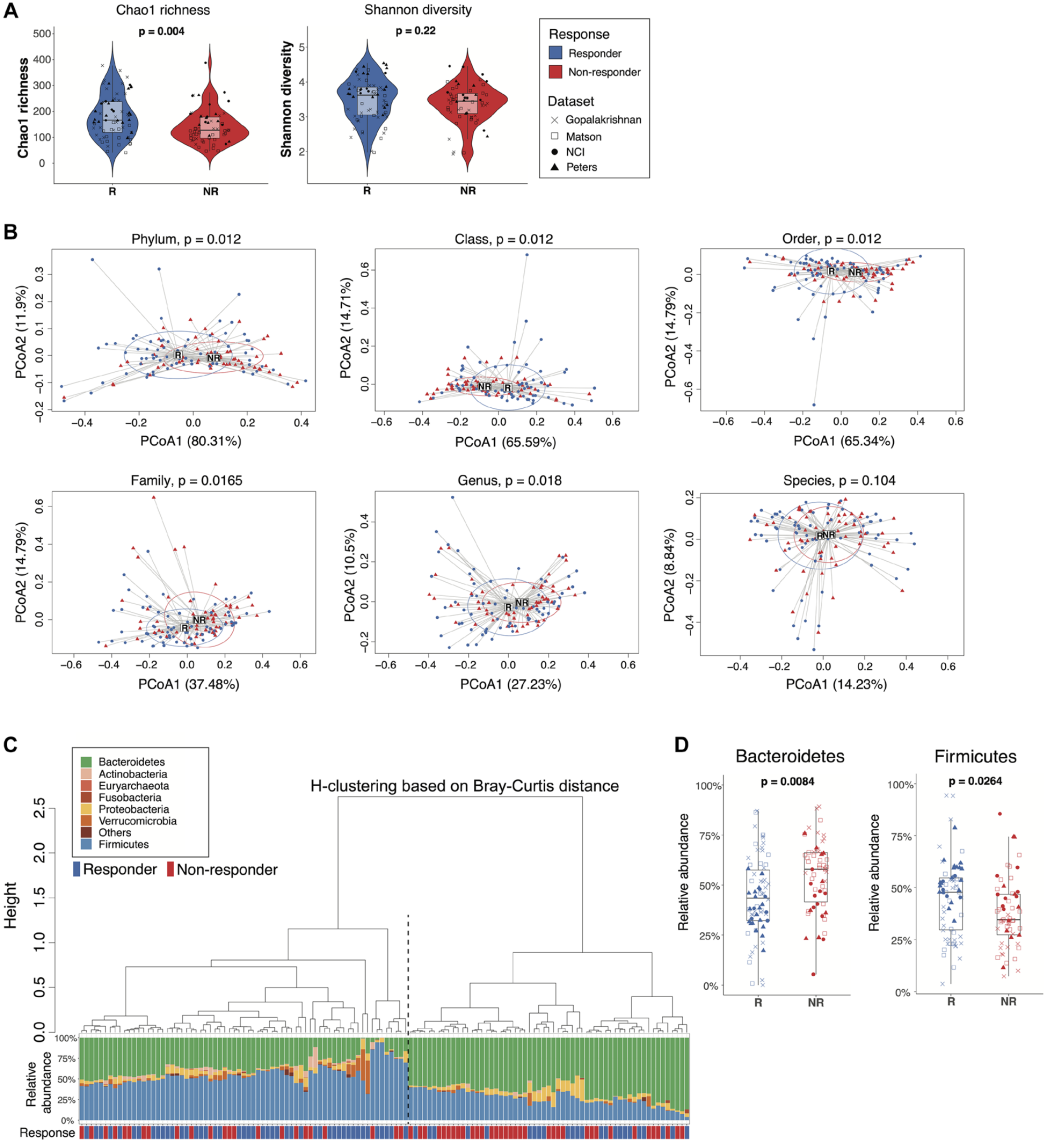
Comparisons of gut microbiome between responders and non-responders from the combined dataset. (**A**) Alpha diversity of the gut microbiome from responders and non-responders was compared by Wilcoxon rank-sum test (unadjusted *p* values). Left: Chao1 richness. Right: Shannon diversity. Boxes represent the first and third quartiles. Upper and lower whiskers extend from the box hinge to the largest/smallest value no further than 1.5^*^IQR. (**B**) Betadisper permutest plots with Bray-Curtis distance from phylum to species level (top left to bottom right) showing the centroid points and distribution of responder and non-responder sample groups. *P* values were adjusted with FDR correction. (**C**) Agglomerative hierarchical clustering of all patient samples using Ward’s method with Bray-Curtis distances at the phylum level. Stacked bar plot shows the relative abundances of bacterial phyla for individual patients. Black dotted line separates cluster 1 (higher response rate) from cluster 2 (lower response rate) (*p* = 0.003, 2-sided proportion z-test). (**D**) Boxplot of selected taxa with differential relative abundance between responders and non-responders (Wilcoxon rank-sum test, *p* < 0.05, FDR-corrected).

To expand the potential generalizability of cancer immunotherapy gut microbiome analyses, we validated our findings using three additional published immunotherapy microbiome datasets from melanoma patients that used a similar sequencing strategy (16S rRNA, V4 region) and comparable response status reporting: Matson et al., [16], Gopalakrishnan et al., [14], and Peters et al., [18] (Supplementary Table 6). The combined dataset was comprised of 128 patient samples (70 responders and 58 non-responders), yielding a total of 5654 unique ASVs (Supplementary Figure 3, Supplementary Tables 7–9). While Chao1 richness was statistically significantly higher in responders as compared to non-responders (*p* = 0.0041, Wilcoxon rank-sum test), Shannon diversity did not differ significantly (*p* = 0.2200, Wilcoxon rank-sum test) (Figure 2A). All 4 datasets were evenly dispersed in the first and second dimensions of NMDS, suggesting that these datasets can be analyzed together due to a lack of study-specific clustering (Supplementary Figure 4).

Analysis of the combined dataset showed statistically significant community differences between the microbiomes of responders and non-responders from phylum to genus levels (Figure 2B). Unsupervised hierarchical clustering of the ASV table based on Bray-Curtis distance at the phylum level clustered patients into two groups with significantly different response rates (cluster1: 0.67, cluster 2: 0.41, *p* = 0.0030, 2-sided proportion z-test) (Figure 2C). Patients in cluster 1 with higher relative abundances of Firmicutes had higher response rates, while patients in cluster 2 were enriched in Bacteroidetes and had lower response rates. Hierarchical clustering at the genus level showed a similar pattern of two clusters of patients with different response rates though not statistically significantly different (cluster1: 0.44, cluster 2: 0.62, *p* = 0.0520, 2-sided proportion z-test) (Supplementary Figure 5). To further evaluate the robustness of these findings, we used Unifrac distance analysis to account for the phylogenetic relationships between taxa. At the genus level, unweighted Unifrac distance, which takes into consideration only the presence or absence of taxa, failed to distinguish responders from non-responders by hierarchical clustering (*p* = 0.0830, 2-sided proportion z-test) (Supplementary Figure 6A). However, weighted-Unifrac, which accounts for relative abundance differences, showed two significantly different hierarchical clusters (*p* = 0.0150, 2-sided proportion z-test) (Supplementary Figure 6B).

Because Bacteroidetes and Firmicutes have previously been associated with immunotherapy response rates [12, 13, 19], we performed a targeted analysis of individual phyla from the combined dataset, which demonstrated a statistically significantly higher relative abundance of Bacteroidetes in non-responders and Firmicutes in responders (Figure 2D). A logistic model predicting responder status based on the relative abundances of Bacteroidetes and Firmicutes yielded an ROC curve with an estimated AUC value of 0.67, suggesting moderate prediction accuracy based on these two phylum level signals alone (Supplementary Figure 7). Thus, microbiome analyses of the combined dataset distinguished responders from non-responders and provided insight into taxa associated with these differences.

### Variable selection identifies microbial signatures most closely linked to response status

**Figure 3:**
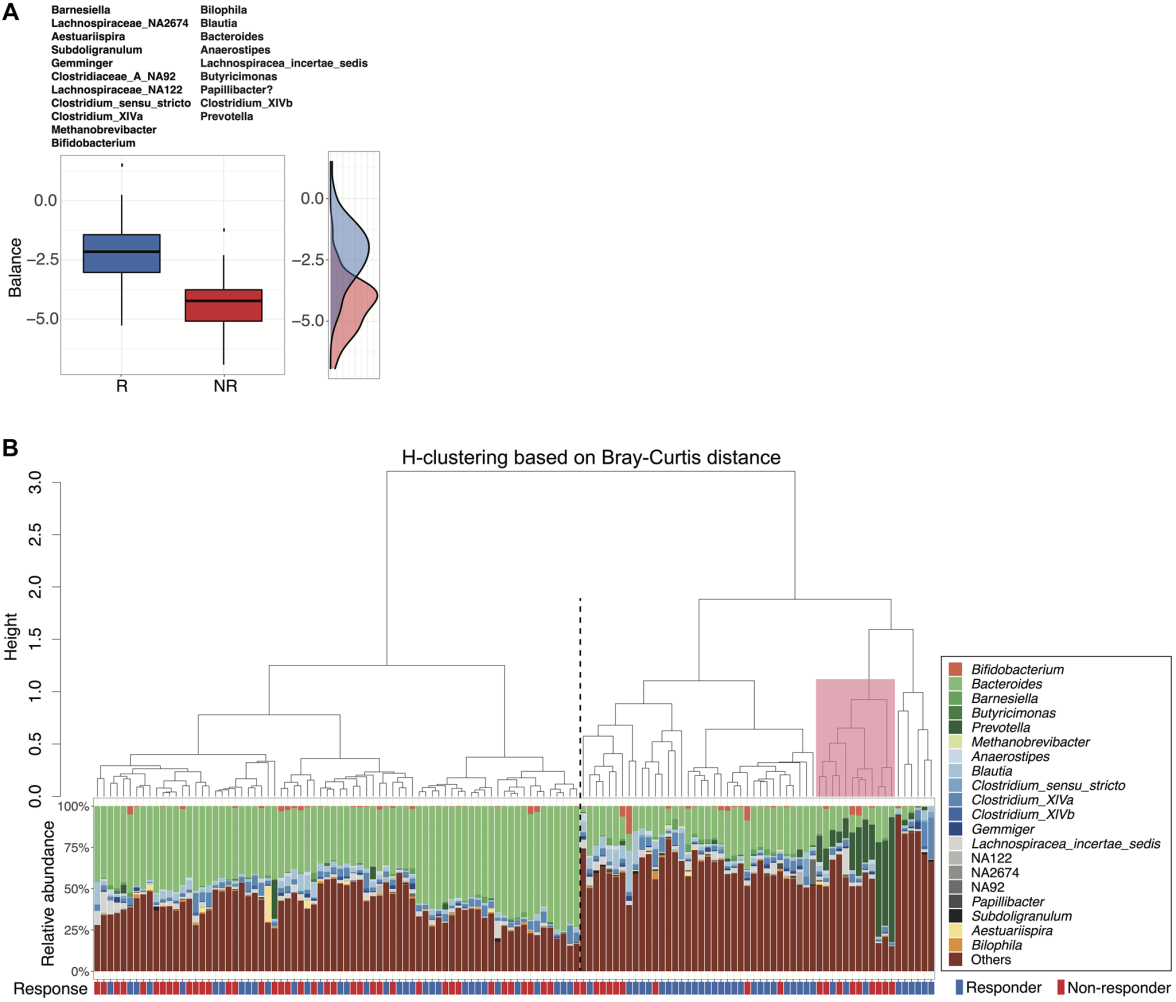
Microbial signatures associated with response status as determined by selbal variable selection. (**A**) Boxplots represent the distribution of balance scores in responders versus non-responders. The balance reflects the compositional difference between the two groups of genera selected by *selbal*. Specific genera enriched in each group are listed above the boxplots. A density plot of balance scores for each group is shown on the right. (**B**) Agglomerative hierarchical clustering of all patient samples using Ward’s method with Bray-Curtis distances calculated from *selbal*-selected genera. Stacked bar plot shows the relative abundances of the selected genera for individual patients. Black dotted line separates cluster 1 (lower response rate) from cluster 2 (higher response) (*p* = 0.02, 2-sided proportion z-test). Pink box highlights a small group (primarily non-responders) within cluster 2 with higher relative abundance of *Prevotella*.

To look beyond individual taxa and more deeply examine groups of taxa (microbial signatures) associated with response status, we utilized *selbal*, an algorithm-based modeling and variable selection method to identify microbial signatures that distinguish responders from non-responders [37]. Of the 20 *selbal*-selected genera, 11 were enriched in responders, while the remaining 9 genera were overrepresented in non-responders (Figure 3A). When analyzed by univariate tests, 7 of the 20 selected genera showed statistically significant differences in relative abundance between responders and non-responders based on unadjusted *p* values but were not statistically significant after FDR correction (Supplementary Figure 8, Wilcoxon rank-sum test), suggesting that *selbal* provides the advantage of identifying certain taxa that may collectively have associations with response status but would not otherwise be identified through univariate analyses. Among these 7 genera, *Bacteroides,* *Bilophila* and *Blautia,* which were overrepresented in non-responders, have been reported in prior studies as enriched in the gut microbiomes of non-responders or patients with shorter progression-free survival (PFS) [14, 18]. *Barnesiella*, Lachnospiraceae_NA2674, *Subdoligranulum*, and *Aestuariispira* were enriched among responders. Consistent with prior studies, we also identified *Bifidobacterium* and *Prevotella* as important predictors of response status using *selbal*, though not statistically significant in univariate analyses [16, 18]. Hierarchical clustering based on Bray-Curtis distances calculated using *selbal*-selected genera clustered patients into two groups with significantly different response rates (cluster1: 0.46, cluster 2: 0.67, *p* = 0.0200, 2-sided proportion z-test) (Figure 3B). A small group of non-responders within cluster 2 had higher relative abundance of *Prevotella* (Figure 3B), suggesting a potential association between *Prevotella* and poorer outcomes.

### Gut microbiome feature modeling predicts clinical response status

**Figure 4:**
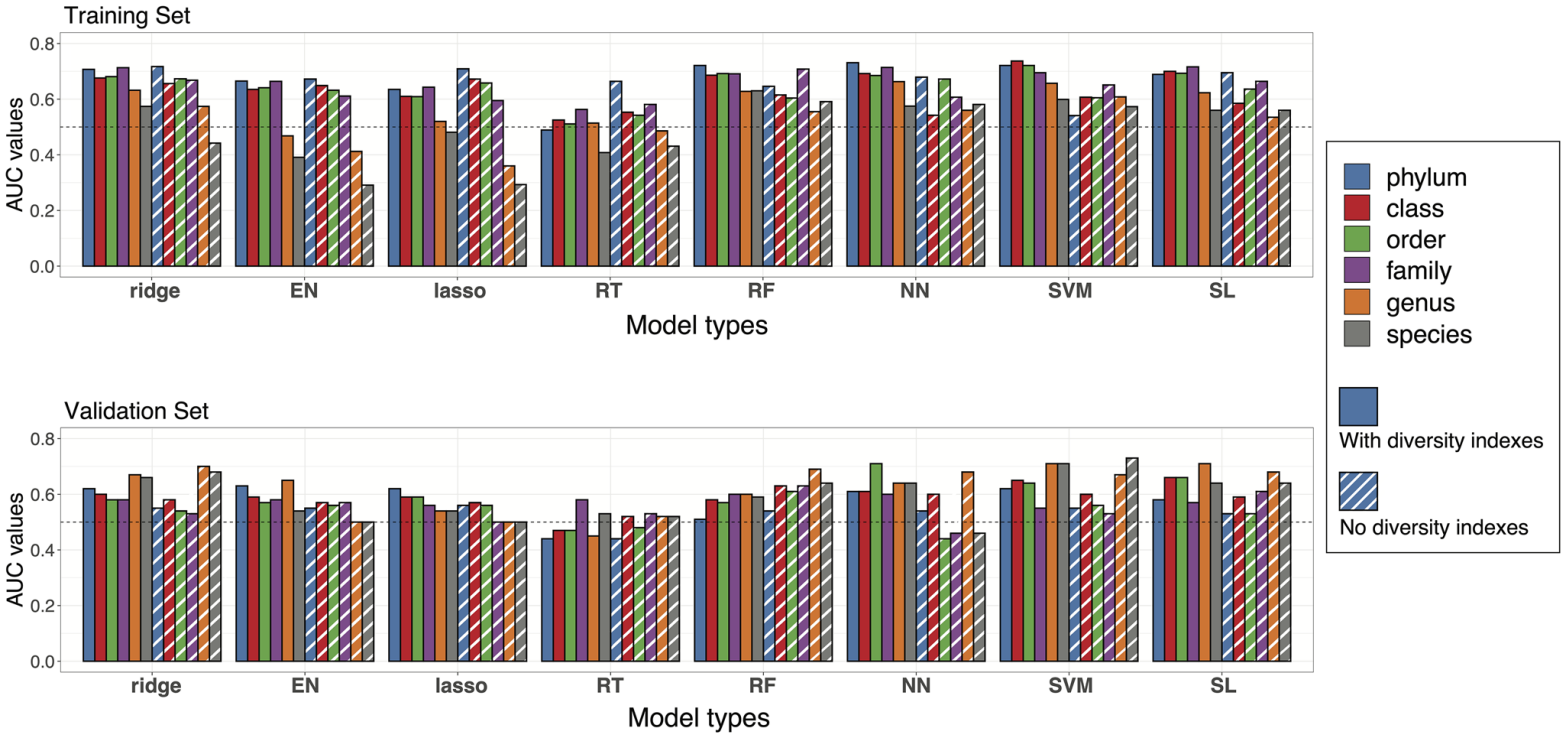
Prediction accuracies of statistical models. Performance of the models at different taxa levels with or without diversity indexes were evaluated by estimated AUC values of ROC curves. Bar plots of AUC values are reported with colors representing different taxa levels and stripes representing the models without diversity indexes. Upper panel: AUC values from the training set in cross validation. Lower panel: AUC values from model testing in the validation set.

We next sought to develop models using microbiome features as clinical response predictors. We included as predictors relative abundances of taxa (species to phylum levels) after removing rare microbes as well as 10 alpha diversity indexes selected to comprehensively represent the community diversity characteristics. The majority of these indexes were weakly correlated with each other based on a Spearman correlation study (Supplementary Table 10, Supplementary Figure 9). Samples from the four combined datasets were randomly divided into a training set (*n* = 88) and a validation set (*n* = 40). To identify the model with the highest prediction accuracy, we tested a large number of diverse machine learning algorithms during model training, including penalized logistic regression with ridge, lasso or elastic net (EN, alpha = 0.5), regression tree (RT), random forest (RF), neural network (NN), support vector machine (SVM), as well as the SuperLearner (SL) ensemble-based algorithm. Prediction accuracy of all models was measured and compared across taxonomic levels using the ROC curve and AUC value. Across all models, AUC values generally increased at higher taxonomic ranks (family and above) (Figure 4, Supplementary Table 11). The inclusion of alpha diversity indexes as predictive features led to a modest, though not statistically significant increase (0.042, two-sided *P* = 0.062, Supplementary Figure 10) in the average AUC (across algorithms and taxa levels). The best prediction accuracy was attained at the class rank with alpha diversity indexes using SVM (AUC = 0.737). Without diversity indexes, ridge regression at the phylum level yielded the best prediction (AUC = 0.717).

Next, we evaluated the accuracy of the prediction equations derived from the training set for predicting tumor response in the validation samples. The AUC estimates in this external validation tended to be lower than those from the internal cross-validation but remained higher than 0.5 (AUC for a purely random assignment) in most cases (Figure 4, Supplementary Table 12). AUC estimates were generally higher when alpha diversity indexes were added to taxa signals as additional features for prediction, but the difference was not statistically significant. The highest AUC estimates were 0.71 and 0.73 for predictions made with or without alpha diversity indexes, respectively. Interestingly, the models generally performed better at lower taxonomic levels (genus/species) in the validation dataset rather than at the phylum or class level.

### Cross-platform validation confirms predictive capacity of the models across sequencing platforms

Because shotgun metagenomic sequencing is an additional method used for analyzing gut microbiomes, we further investigated whether the prediction equations generated with the training set of 16S rRNA V4 sequencing data would predict responses in two independent shotgun metagenomic sequencing datasets from melanoma patients: Frankel et al., [13] (19 responders and 20 non-responders) and McCulloch et al., [38] (19 responders and 8 non-responders). To generate comparable taxonomy variables as the 16S rRNA amplicon sequencing data, we included relative abundances of family to phylum level taxa as features without alpha diversity indexes (Supplementary Table 13). We tested our prediction equations on the two shotgun metagenomic datasets both individually and jointly. AUC estimates were generally higher with McCulloch et al., data, but the difference in prediction accuracy was not statistically significant between datasets (Supplementary Figure 11, Supplementary Table 14). The best prediction accuracy was attained at the order level with the RF algorithm (AUC = 0.73) when testing with McCulloch et al., dataset. The highest AUC estimates for the Frankel et al., dataset and the combined datasets were 0.67 and 0.63, respectively, revealing a weaker yet still meaningful prediction accuracy. Thus, statistical models utilizing gut microbiome community features were robust to a different sequencing platform.

## DISCUSSION

In this study, we identified baseline microbiome features associated with immunotherapy response based on a combined analysis of three previously published 16S rRNA gene sequencing datasets from melanoma patients along with our additional cohort of advanced stage solid tumor patients enrolled in NCI immunotherapy trials. By using a tumor-agnostic approach for the discovery cohort and by analyzing these data in combination with larger melanoma datasets, we uncovered microbiome response signals that may be generalizable across tumor types; however, additional investigation with large-scale cohorts of different tumor types will be needed to confirm these findings. In the meta-analysis, we found that responder microbiomes more clearly separated from non-responder microbiomes at higher taxonomic levels. Whereas previous studies have primarily focused on individual taxa associated with immunotherapy response, we examined more complex community-based interactions using *selbal* analysis and uncovered differentially abundant groups of taxa (i.e., microbial signatures) that associated with clinical response status. To predict immunotherapy response, we developed and validated statistical models using taxonomic variables and community diversity indexes as predictive features. These models had good prediction accuracy in 16S training and validation datasets and, importantly, maintained predictive capacity across sequencing platforms when verified separately on shotgun metagenomic sequencing datasets.

Although several previous studies have identified potential microbiome-based biomarkers for cancer immunotherapy response, prior investigations have primarily focused on melanoma patients and largely inconsistent taxa. Different studies have reported higher relative abundances of *Faecalibacterium*, *Bifidobacterium* or *Akkermansia* in responders [12–14, 16, 18, 19], and *Bacteroides* or Bacteroidales in non-responders [12, 14, 18], yet, no clear consensus signals have emerged. In our meta-analysis, we accounted for multiple variables that could contribute to the inconsistencies observed across studies by geographically restricting our analyses to US-based patient cohorts with consistent responder/non-responder classification; by including only datasets that used comparable sequencing techniques (16S V4 region); and by re-analyzing previously published datasets using one standardized pipeline. When we initially analyzed the NCI cohort alone, we took an agnostic approach to include multiple tumor types to find common microbiome features. A few bacterial genera were identified as significantly enriched among responders (e.g., *Ruminococcus2* and *Eggerthella)*, and some previously reported genera (*Prevotella* and *Akkermansia*) were also differentially abundant between responders and non-responders but not statistically significant, possibly due to the small sample size [14, 19]. In the meta-analysis, we further validated the identified common microbiome features with larger datasets from melanoma patients. Responder microbiomes separated more distinctly from non-responder microbiomes when analyzed at higher taxonomic levels, indicating that taxonomic rank may be an important variable to consider when searching for microbiome features consistently associated with response status. The lack of significant separation between responders and non-responders at lower taxonomic levels could reflect a prevalence of low abundance species or genera that are not adequately resolved by the sequencing methodology. This could also result from heterogeneity in the patient population and inter-individual variation between subjects, which may be more apparent at lower taxonomic ranks. By using unsupervised hierarchical clustering at the phylum level, we observed a higher response rate in patients enriched in Firmicutes and a lower response rate in patients enriched in Bacteroidetes. Interestingly, recent systemic antibiotic usage has been associated with an increase in the Bacteroidetes/Firmicutes ratio and linked to poorer immunotherapy outcomes [35, 36, 39]. These findings align with the microbiome features associated with non-responders in our analyses.

While individual taxa may strongly influence biological outcomes, we sought to identify more complex microbial community interactions that might improve prediction of immunotherapy response. Thus, we analyzed our combined dataset using *selbal*, a newer method suitable for finding differentially abundant groups of taxa (microbial signatures) in compositional data [37, 40]. *Selbal* analyses identified two groups of genera associated with responders or non-responders, which included several genera discussed in previous publications (i.e., *Bacteroides*, *Bifidobacterium*, *Clostridium*_XIVa, *Blautia* and *Gemmiger*). Importantly, most of the *selbal*-selected taxa were not initially identified using univariate analyses, highlighting the relevance of multivariate analyses in identifying taxa groups, which may collectively associate with response status through direct or functional interactions.

To predict immunotherapy response status, we developed and validated statistical models using global microbiome features and major taxonomic variables. Using 16S data, our training and validation sets demonstrated a favorable prediction accuracy with the highest AUC value around 0.75, which is equal to or higher than the prediction accuracy reported for several previously published microbiome-based models of immunotherapy response [41–43]. Our models achieved this level of prediction accuracy based solely on the inclusion of taxonomic features and diversity indexes, and they outperformed some previous models that included both taxonomic features and metagenome-derived functional features (e.g., Kyoto Encylopedia of Genes and Genomes/KEGG ortholog differential abundances) [41, 43]. Model prediction accuracy varied substantially across taxonomic levels. Interestingly, taxonomic rank appeared to have a greater effect on prediction accuracy than the chosen machine learning algorithm, indicating that taxonomy level may be an important driver in model performance. Because the inclusion of functional features generally increased model performance in prior studies, the prediction capacity of our models may be even further enhanced by the future inclusion of functional features. Strikingly, our models were additionally validated on microbiome data generated using a shotgun metagenomic sequencing platform with one of two shotgun datasets yielding a robust prediction accuracy. Multiple factors including the technical differences of distinct sequencing platforms and batch effects from different experiments likely affected the effectiveness of the models. However, the cross-platform applicability of our models is encouraging and suggests the possibility of broader generalizability across disparate datasets.

Our study serves as an example of how small patient cohorts can be appropriately integrated into a larger body of published data to elucidate biologically meaningful conclusions. Prior efforts to optimize consistency between studies have focused on matching potential confounding variables between cases and controls at the study design stage [44]. This remains an ideal approach yet may preclude the use of publicly available sequencing data. Based on our meta-analysis, there are several important parameters to consider in future studies to effectively combine distinct datasets for the consistent identification of gut microbiome features associated with disease states or treatment response. These factors include careful selection and inclusion of samples from the following: patients with equivalent classification of disease state or treatment response; patient cohorts from comparable geographic regions; patients with appropriately annotated clinical metadata; datasets generated using equivalent sequencing regions/technologies; and datasets processed and re-analyzed using a single standardized pipeline. Our meta-analysis provides a model for combining distinct datasets in a controlled manner; this methodology may enable biological findings from large-scale analyses to be extrapolated and applied to size-limited patient cohorts in future studies.

Our study is limited by the sample size. While our findings suggest that certain immunotherapy response signals may be generalizable across different tumor types, this must be confirmed in larger patient cohorts. Large-scale investigations are needed to distinguish potential tumor-agnostic response signals from those that may be associated with specific tumor types. An additional limitation of this study is the resolution of our sequencing data when analyzing at lower taxonomy levels. We utilized an ASV clustering method at species and genus levels, and the bacterial variables generated by this method cannot be applied to other sequencing methods. This problem could be rectified by using long read 16S sequencing methodologies, which would likely improve classification accuracy at lower taxonomic levels [45, 46]. The performance of our models may be potentially enhanced by the inclusion of additional clinical variables such as antibiotic usage, body mass index, transcriptomic or proteomic data. The KEGG pathways or meta-transcriptomics features from gut microbiome have been analyzed by several research groups to identify the different patterns between the microbiome of responders and non-responders [18, 27, 29, 42, 43]. Thus, additional microbiome features may be beneficial for future model development.

In conclusion, analyses of our cohort and the combined microbiome dataset have provided a robust assessment of immunotherapy patients’ gut microbiomes. The development of reliable models provides additional opportunity to distinguish and predict immunotherapy responders from non-responders. However, the interactions between key microbial taxa and host immunity still need to be elucidated. Ultimately, this research will assist in identifying microbial biomarkers or novel therapeutic targets to improve immunotherapy outcomes and the overall survival of cancer patients.

## MATERIALS AND METHODS

### NCI patient cohort and sample collection

Adult oncology patients who were enrolled in National Cancer Institute cancer immunotherapy clinical trials (NCT01772004, NCT02517398, NCT02496208) at the National Institutes of Health Clinical Center in Bethesda, MD USA were recruited to participate in gut microbiome sample collection through IRB-approved protocols (NCT00001506, NCT02471352) from 2014 to 2018. Any subject willing to participate and provide stool samples were eligible. All patients enrolled in this study provided written informed consent. Demographic, medical treatment and clinical information are summarized in Supplementary Table 1. Radiographic response to therapy while receiving immunotherapy was determined by treating investigators and assessed using response evaluation criteria in solid tumors (RECIST) v1.1. Subjects classified by RECIST criteria as having complete response (CR), partial response (PR), or stable disease (SD) for at least 6 months were considered responders. Subjects with progressive disease (PD) or stable disease for less than 6 months were considered non-responders. Response status reflects the best overall response from the start of treatment until disease progression or the end of the study. Mean age between responders and non-responders was compared using Student’s *t*-test. Subjects provided stool samples prior to starting immunotherapy trials. Samples were immediately stored in −80°C freezer until processing.

### DNA extraction, PCR amplification, and sequencing

Stool DNA was extracted following the PowerSoil DNA Isolation Kit protocol (Qiagen Cat no. 47014). Final DNA concentration was measured by Qubit BR DNA Kit. Extracted DNA was used for PCR amplification of the 16S rRNA gene V4 region with 515F/806R primer pair (515F: 5′-GTGCCAGCAGCCGCGGTAA-3′, 806R: 5′-GGACTACCAGGGTATCTAAT-3′), which contains adapters for Illumina MiSeq sequencing and sample-specific barcodes. PCR reactions were performed in 30 cycles. PCR products were pooled in equimolar amounts before purification with AMPure xp magnetic beads and then quantified with Qubit BR DNA Kit. Sequencing was performed on the NGS MiSeq platform (Illumina, Inc, San Diego, CA, USA) at NIH Intramural Sequencing Center or NIH Laboratory of Integrative Cancer Immunology, Microbiome and Genetics Core with reagent, sequencing, and collection controls used to test for background and collection contamination. 2 × 250 bp paired end MiSeq run was used to yield forward and reverse reads with close to full overlap.

### Sequencing read analyses and taxonomy profiling

Raw sequencing reads were analyzed using the DADA2 pipeline (version 1.16.0) in R (version 4.0.5) to generate exact amplicon sequencing variants (ASV) based on the guidelines at https://benjjneb.github.io/dada2/tutorial.html [47]. Briefly, raw sequences were processed to remove barcodes and quality filtered with default settings (maxN = 0, maxEE = c(2,2), truncQ = 2, rm.phix = TRUE). Based on the quality scores and for the purpose of maximum merging, forward and reverse reads were truncated to be 180 bp and 139 bp and followed by primer trimming. Dereplicating, merging, and chimera removal were processed with default settings. After processing, a seqtab table was generated with rows corresponding to samples and columns corresponding to ASVs.

For taxonomy profiling, ASVs from the seqtab table were aligned against the RefSeq-RDP reference database (RefSeq-RDP16S_v2_May2018.fa.gz) to generate the taxonomic table assigned to the species level [48]. ASVs which failed to assign at species and genus levels were clustered by sequence similarity to species/genus-level operational taxonomic units (OTU) by kmer package (version 1.1.2) in R (version 3.6.1). Thresholds for clustering were set to 0.99 (for species-level OTUs) and 0.97 (for genus-level OTUs). Output OTUs and representative sequences were combined with assigned species or genera to form the final taxonomic table. For taxonomic tables at family or upper levels, un-assigned ASVs were removed from downstream analyses.

### Microbiome community analyses

Read counts from the taxonomic table were converted into relative abundances by dividing the count of each taxon by the sum of read counts within each sample. Alpha diversity indexes were calculated with vegan package (version 2.5.7) or microbiome package (version 1.8.0), and Bray-Curtis dissimilarity distances were calculated via vegan package in R. Both alpha and beta diversity calculations were based on ASV-level relative abundances. Bar-plots and boxplots to visualize the microbiome community difference between responders and non-responders were generated with genus-level relative abundances. To reduce the number of rare microbes and multi-testing, only the taxa with more than 0.1% relative abundance in at least 10% of samples at each taxonomy level from species to phylum were included in the volcano plot. Wilcoxon rank-sum tests with unadjusted as well as false discovery rate (FDR)-corrected *p*-values were used to determine the taxa with significantly different relative abundance (*p* < 0.05) between responder and non-responder samples.

### Literature review and published dataset collection

PubMed was searched for published literature relating to gut microbiome-immunotherapy interactions to find microbiome sequencing data, particularly datasets of 16S rRNA gene sequencing of the V4 region. Based on consideration of sequencing region/techniques and availability of sequencing datasets and patient metadata, 3 eligible studies from Matson et al., [16], Gopalakrishnan et al., [14], and Peters et al., [18] were selected for statistical model development. Sequencing datasets were downloaded from the Sequence Read Archive (SRA) with the accession numbers PRJNA399742 for Matson et al., PRJEB22894 for Gopalakrishnan et al., and PRJNA541981 for Peters et al., Patients’ response information was obtained from the study GitHub repository (Matson dataset) (https://github.com/cribioinfo/sci2017_analysis), the SRA data library website (Peters dataset), or kindly received from the authors (Gopalakrishnan dataset).

### Response status of pooled samples

Response status after immunotherapy was consistently assigned for patients from selected datasets. Responder and non-responder groups were classified according to RECIST 1.1 based on available metadata. For NCI and Gopalakrishnan datasets, patients were classified as responders if they achieved CR, PR, or SD with PFS no less than 6 months. Patients with PD, or SD with PFS less than 6 months were considered non-responders. For Matson et al., in which PFS information is not available, patients with SD were treated as non-responders according to the publication [16]. Patients with PFS no less than 6 months were considered responders in Peters dataset, as only PFS information is reported. In total there are 128 samples, including 70 responders and 58 non-responders.

### Combining sequencing data and taxonomy profiling

Sequencing data were analyzed by DADA2 pipeline as described above. According to the quality scores and pipeline for NCI dataset, forward and reverse reads were truncated to be 140 bp before merging for samples from Matson et al., and Peters et al., Reads for Gopalakrishnan et al., samples were merged already and subsequently truncated to be 254 bp. Dereplicating, merging, and chimera removal were processed with default settings. Seqtab tables with rows corresponding to samples and columns corresponding to ASVs from each DADA2 run were combined into a final seqtab table for taxonomy classification. All ASVs were trimmed to 252 bp and merged by ASV sequence in R during table combining. 5654 unique ASVs were generated after combining all samples from 4 datasets.

ASVs were aligned against the RefSeq-RDP reference database (RefSeq-RDP16S_v2_May2018.fa.gz) to generate the taxonomic table as described above [48]. ASVs that failed to assign at species and genus levels were clustered by the sequence similarity to species/genus-level OTU signals as described above. Output OTU signals and representative sequences were combined with assigned species or genera to form the final taxonomic table. For taxonomic tables at family or upper levels, un-assigned ASVs were removed from downstream analyses.

### Diversity calculation

Alpha diversity indexes were calculated with vegan and microbiome package in R with ASV-level relative abundances. For data standardization (maximum method), raw reads were standardized with decostand function from vegan package (method = “max”). Chao1 richness index was calculated with read counts rarefied to 7378 reads per sample, which is the lowest sequencing depth among all samples. Wilcoxon rank-sum tests were performed to determine the diversity indexes with statistically significant differences (*p* < 0.05) between responders and non-responders.

Beta diversity was assessed based on ASV-level relative abundances using the Bray-Curtis dissimilarity distance calculated via vegan package in R. Non-metric multi-dimensional scaling (NMDS) plot was used to visualize the distance between samples from different datasets.

### Statistical analyses

Permutational multivariate analyses of variance (PERMANOVA) test was performed to study the group difference between responder and non-responder samples by considering both the difference of mean values and within-group variation (dispersion) [49]. Only signals with more than 0.1% relative abundance in at least 10% of samples were included at each taxonomy level from species to phylum (final number of taxa features from species to phylum levels are 169, 83, 25, 15, 13 and 7, respectively). The test was performed with adonis function (vegan package) based on Bray-Curtis distance matrices calculated at each taxonomy level. Betadisper plots were created to visualize the centroids of each group and the distribution of data points in the principal coordinates-derived Euclidean space.

Hierarchical agglomerative clustering (i.e., Ward’s method) was applied to Bray-Curtis distance matrices or Unifrac distances calculated with filtered information at each taxonomy level with agnes function (cluster package, version 2.1.0). Percentage of responders from 2 major patient clusters were compared using 2-sided proportion z-test. A *p*-value of 0.05 was set as the cutoff of statistical significance. *P*-values were adjusted using FDR correction except when specified in the text to be unadjusted.

Relative abundance values of Firmicutes and Bacteroidetes were fit into a generalized linear model by glm function in R. Response type was used as the predictor factor with parameter “family = binomal”. A receiver operating curve (ROC) reflecting model results was generated by rocit function from ROCit package (version 2.1.1) using the empirical method.

### Selection of taxonomy signals

Key genera associated with response types were studied using *selbal* package (version 0.1.0). A forward selection process with 5-fold cross-validation was performed to optimize the taxa variables and maximize the balance score between the two response groups. We used filtered relative abundances at genus level to minimize the number of tests. Variables selected in the global balance table were reported as key genera reflecting microbiome differences between responder and non-responder samples. Boxplot with balance scores was generated from *selbal* analysis output. Boxplots for each selected genera were created in R (version 3.6.1). Wilcoxon rank-sum tests were performed to determine the genera with statistically significant difference (*p* < 0.05) between responders and non-responders using unadjusted and FDR-corrected *p*-values.

### Tumor response prediction using machine learning algorithms

The SuperLearner (SL) package in R was used to make predictions based on the SL and individual algorithms (penalized logistic regression with ridge, lasso or elastic net (EN), as well as regression tree (RT), random forest (RF), neural network (NN), support vector machine (SVM)) via wrapper functions for the glmnet, rpart, randomForest, nnet and svm packages. SL yields an optimized weighted average of the aforementioned algorithms.

The algorithms were evaluated using the training set of 88 patients and internally validated through 10-fold cross validation. The resulting prediction equations were externally validated using the validation set of 40 patients and separate shotgun metagenomic sequencing datasets. A nonparametric bootstrap procedure was used to obtain standard errors for area under the curve (AUC) estimates. For internal cross-validation, the bootstrap procedure was to repeatedly sample 88 patients with replacement from the training set of 88 patients and compute an AUC estimate for each bootstrap sample. In external validations, the prediction equations from the training data were considered fixed, and bootstrap sampling was performed on the validation set of 40 patients or the shotgun metagenomic sequencing dataset.

### Shotgun metagenomic sequencing data analyses

PubMed was searched for publications with available shotgun metagenomic sequencing datasets. We analyzed metagenomic sequencing data reported in the study by Frankel et al., from the SRA with accession number SRP115355 [13] and by McCulloch et al., [38] whose data were made available with accession number PRJNA762360. Reads mapping to human genome database (humanGRCh38) were identified and removed using Bowtie2 and samtools programs. Prinseq was used for trimming of low-quality reads and seqtk further split the output files into forward and reverse reads which went through MetaPhlAn3 pipeline for taxonomic profiling [50]. Taxonomy information was compiled for downstream analyses.

## SUPPLEMENTARY MATERIALS





## Abbreviations

ICIs: immune checkpoint inhibitors
PD-1: programmed cell death protein 1
PD-L1: programmed death-ligand 1
CTLA-4: CTL antigen 4 protein
NCI: National Cancer Institute
ASV: amplicon sequencing variant
NMDS: non-metric multi-dimensional scaling
ROC: receiver operating curve
PFS: progression-free survival
PERMANOVA: permutational multivariate analyses of variance
SL: SuperLearner
EN: elastic net
RT: regression tree
RF: random forest
NN: neural network
SVM: support vector machine
AUC: area under the curve
KEGG: Kyoto Encylopedia of Genes and Genomes
OTU: operational taxonomic units
