# Supplementary material for: Predicting cancer immunotherapy response from gut microbiomes using machine learning models
Source: Oncotarget. 2022 Jul 19;13:876–89. doi: 10.18632/oncotarget.28252 (PMC9295706; doi:10.18632/oncotarget.28252)
Supplement: Supplementary file 1 [file oncotarget-13-28252-s001.pdf]

Predicting cancer immunotherapy response from gut microbiomes using machine learning models

SUPPLEMENTARY MATERIALS

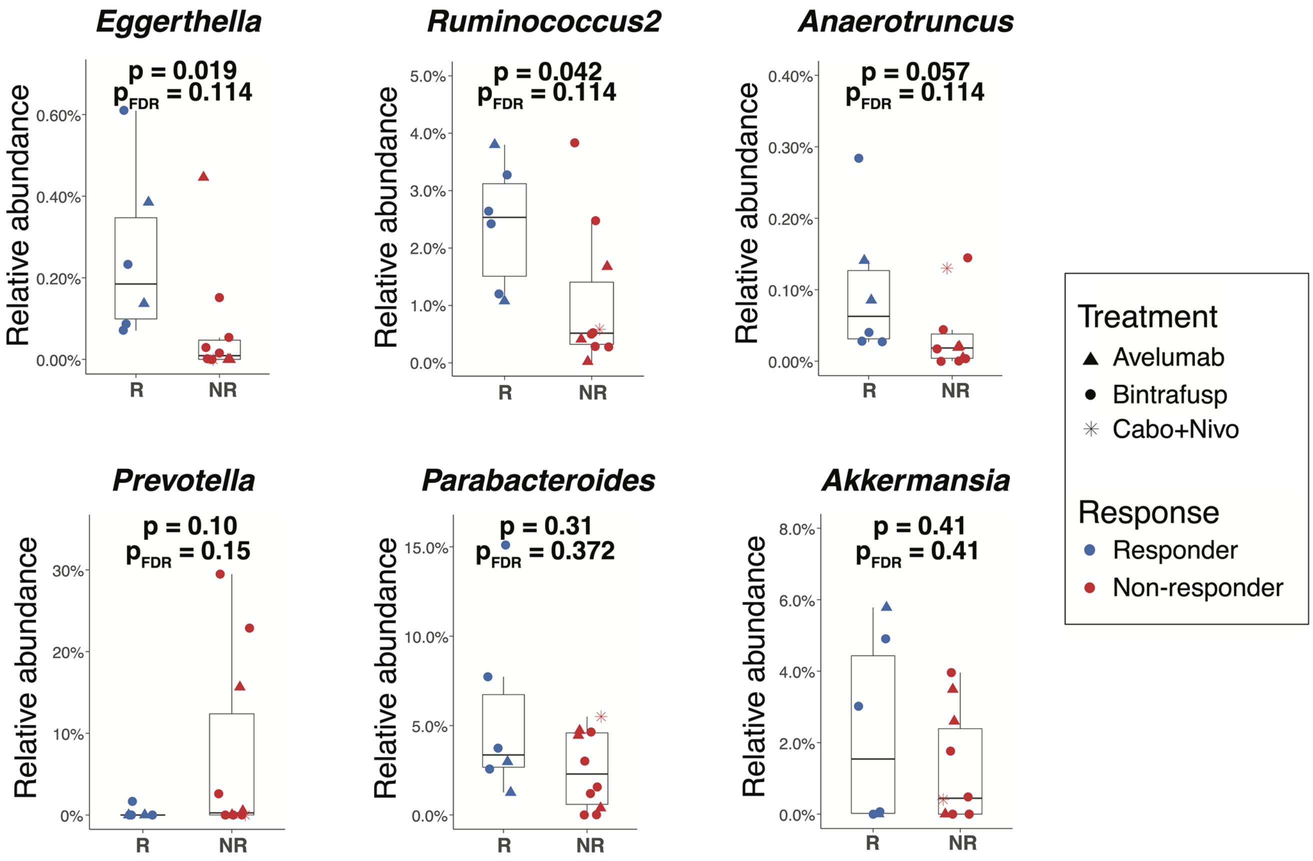

Supplementary Figure 1: Boxplot of selected genera with differential relative abundance between responders and non-responders (Wilcoxon rank-sum test, unadjusted and FDR-corrected *p* values). Boxes represent the first and third quartiles. Upper and lower whiskers extend from the box hinge to the largest/smallest value no further than 1.5*IQR.

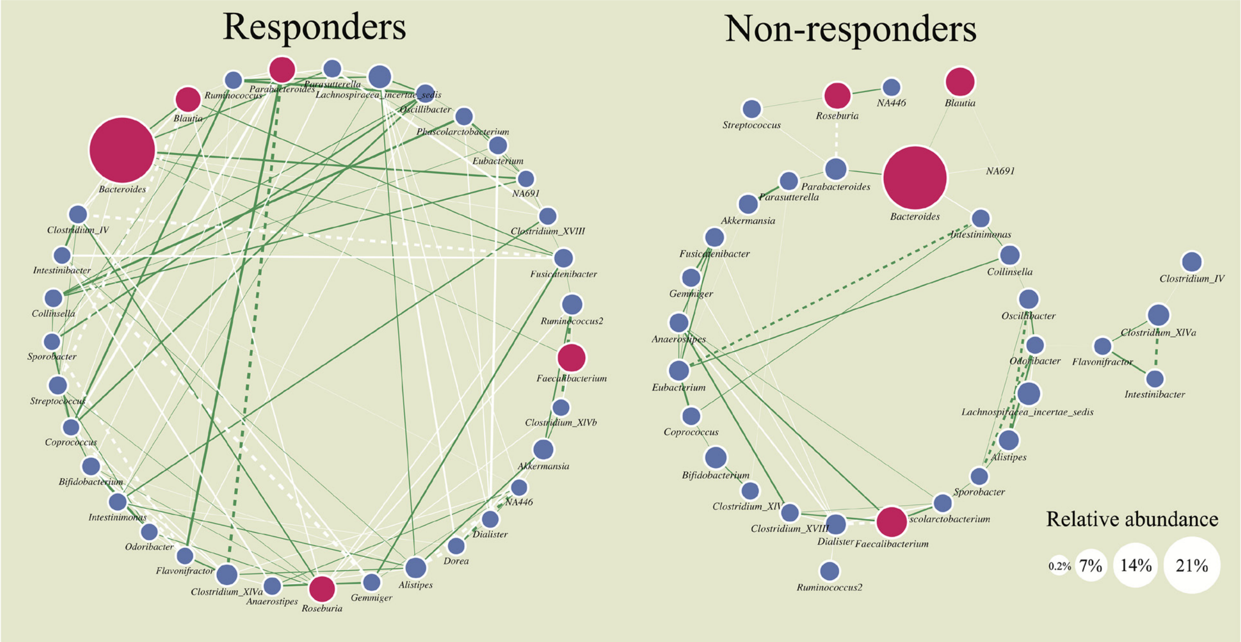

Supplementary Figure 2: Co-occurrence network of human gut bacteria with strong correlations (i.e., correlation coefficient >0.6). The nodes represent bacterial genera with size indicating the mean relative abundance across responders or non-responders in the NCI cohort. Bacteria with relative abundance above 5% are highlighted in red. Green and white lines indicate positive and negative co-occurrence, respectively. Line widths are proportional to the values of the correlation coefficients. Significant correlations (>0.9 for responders and >0.85 for non-responders) are highlighted with dashed lines.

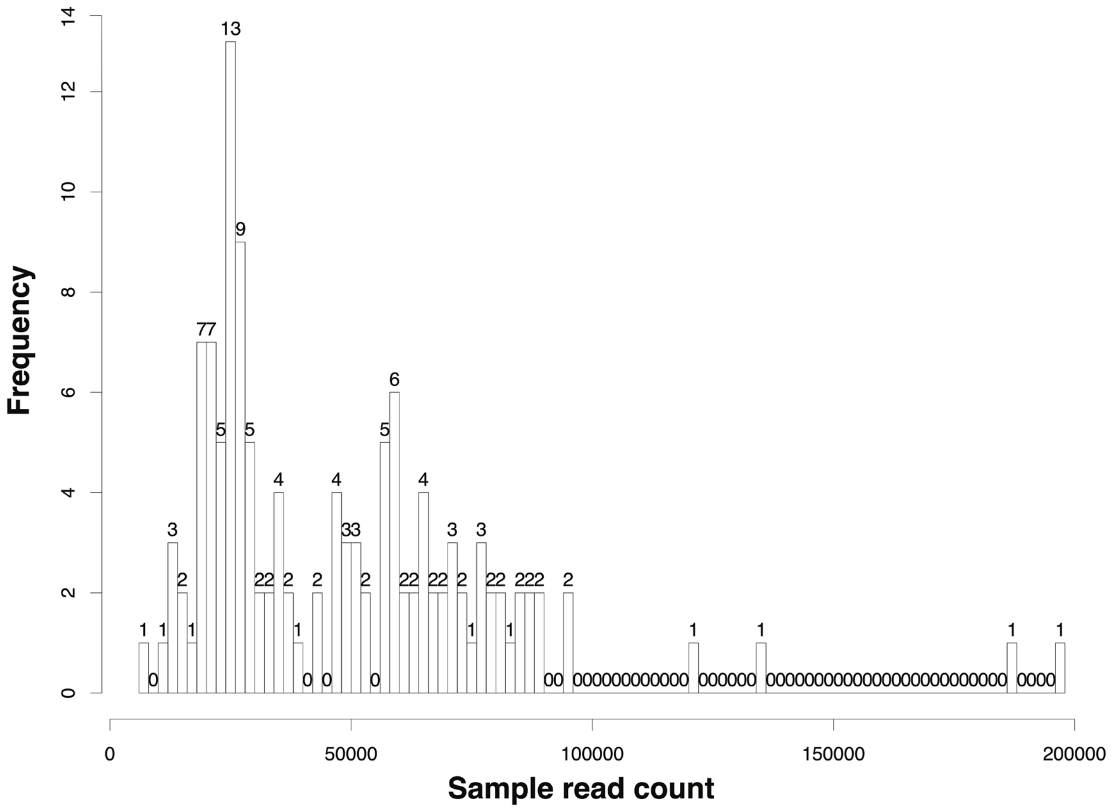

Supplementary Figure 3: Histogram of sample read counts from the combined dataset after merging data from the NCI cohort with 3 published datasets.

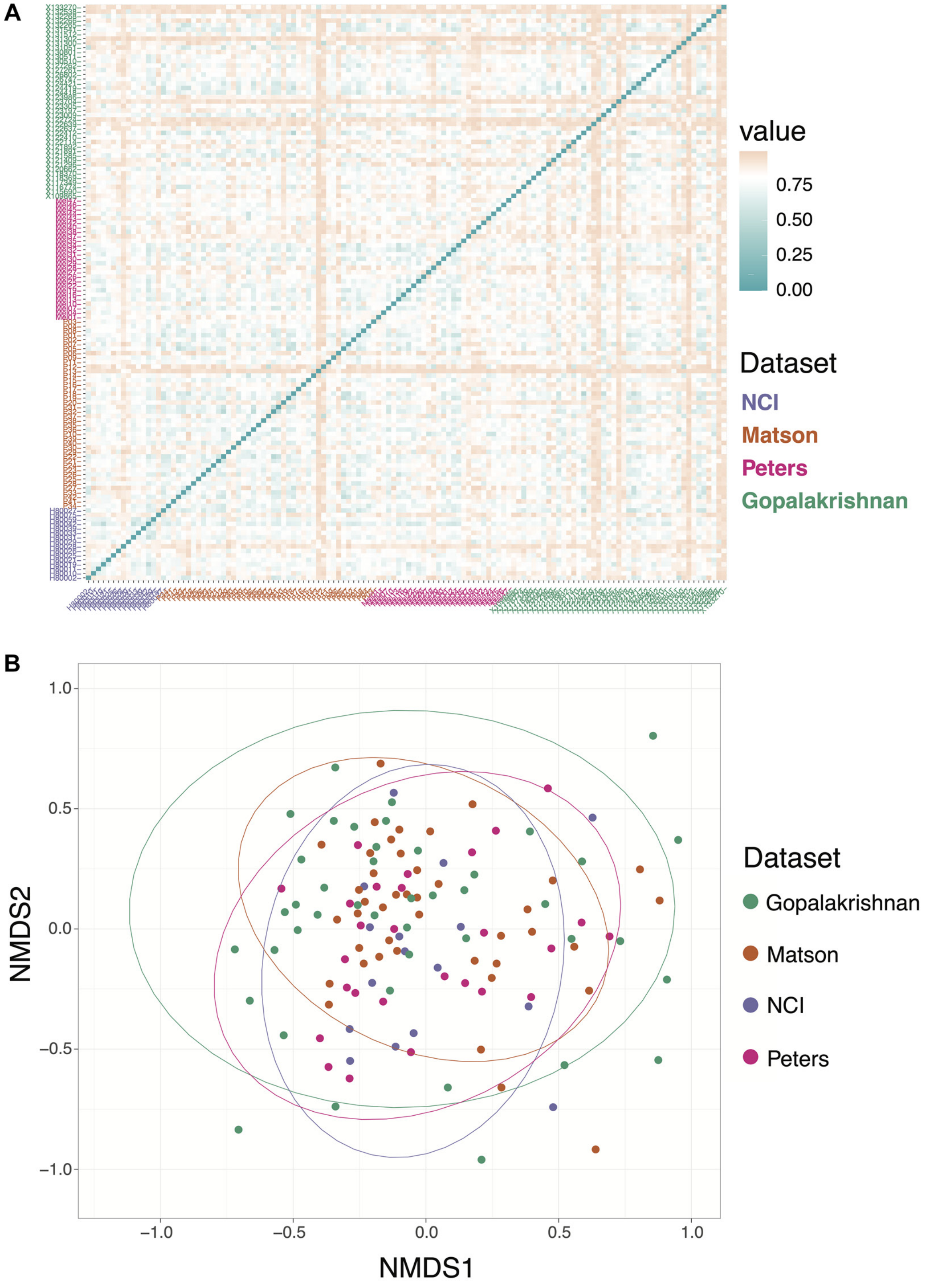

Supplementary Figure 4: (**A**) Heatmap of Bray-Curtis distance matrix at the ASV level among samples from 4 datasets showing a lack of study-specific clustering. (**B**) Non-metric Multi-dimensional Scaling (NMDS) plot with Bray-Curtis distance at the ASV level. Samples are colored by dataset.

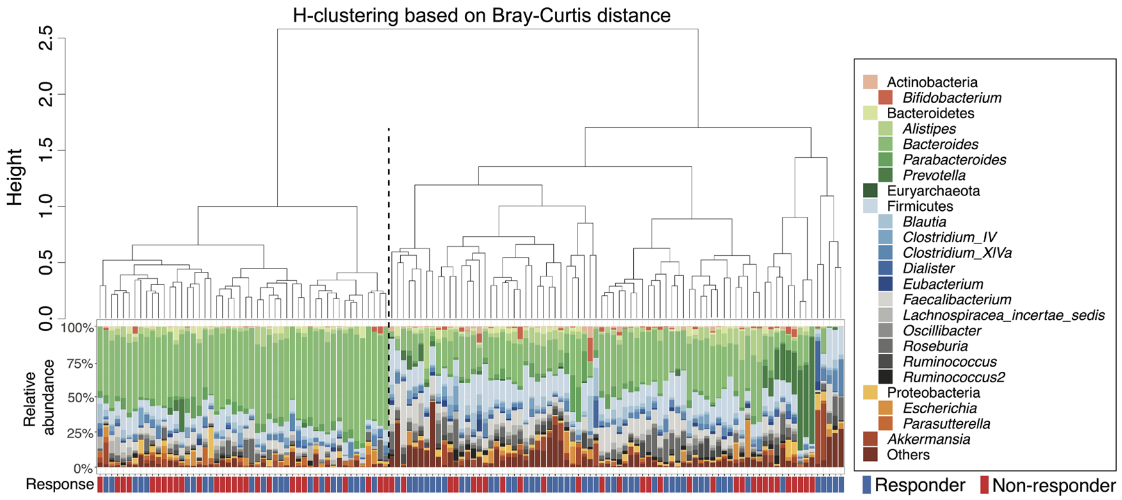

Supplementary Figure 5: Agglomerative hierarchical clustering of all patient samples using ward's method with Bray-Curtis distances at the genus level. Stacked bar plot shows the relative abundances of bacterial genera for individual patients. Black dotted line separates cluster 1 (lower response rate) from cluster 2 (higher response rate) ($p = 0.052$, 2-sided proportion z-test).

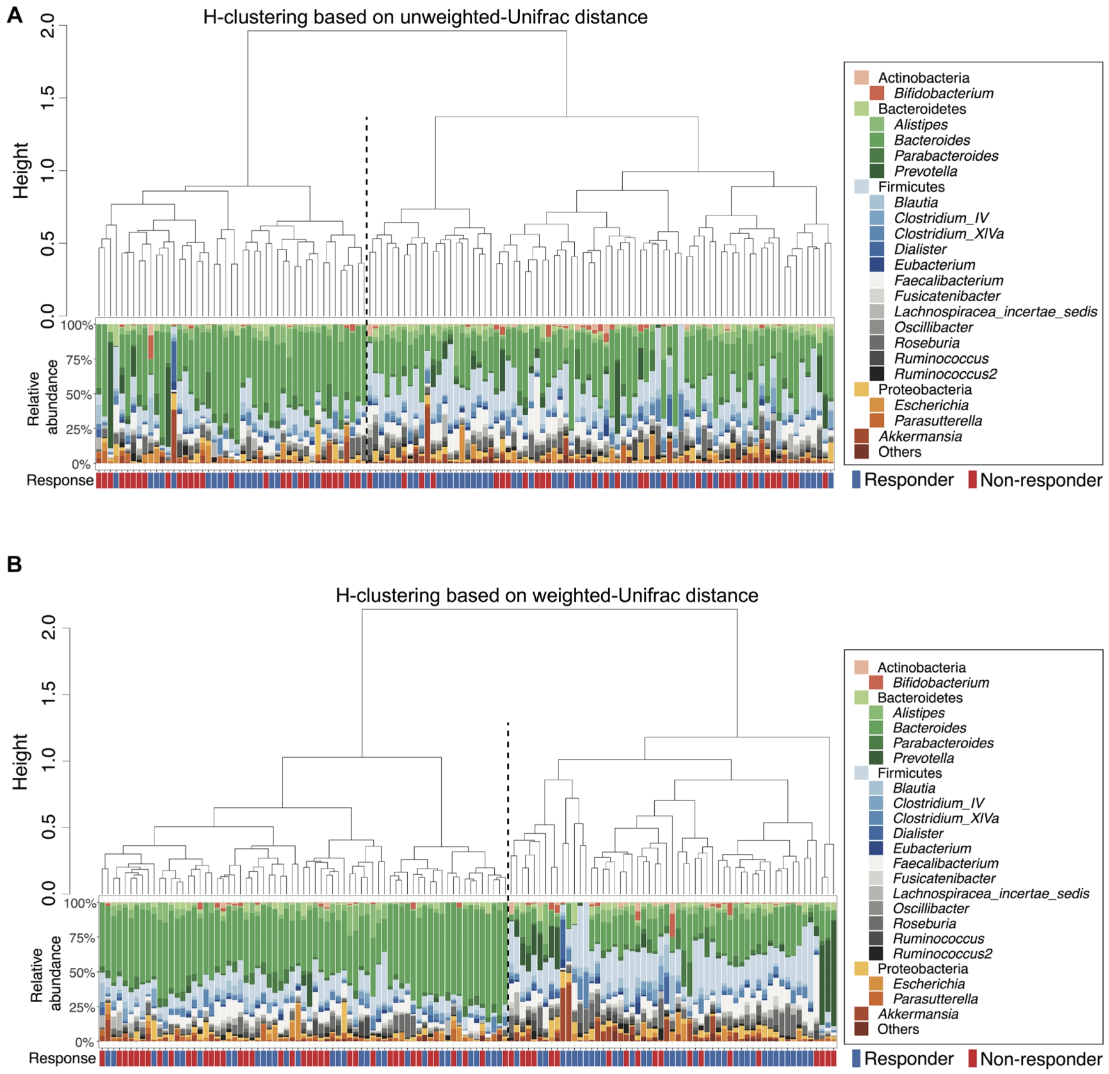

Supplementary Figure 6: Agglomerative hierarchical clustering of all patient samples using ward's method with (**A**) unweighted-Unifrac distance or (**B**) weighted-Unifrac distance at the genus level. Stacked bar plots show the relative abundances of bacterial genera for individual patients. Black dotted lines separate patient clusters.

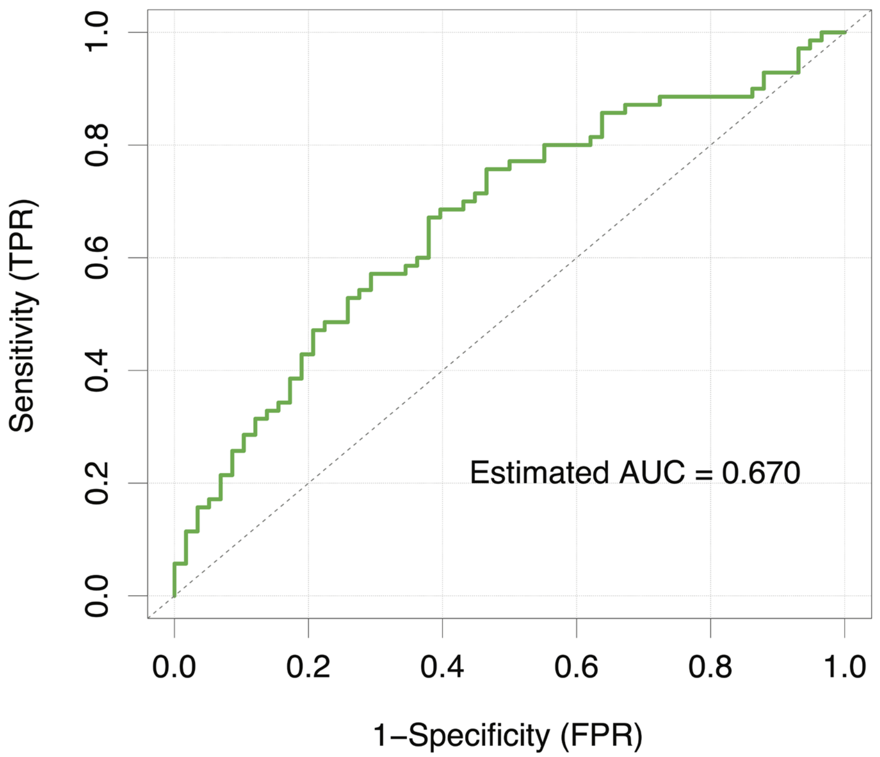

Supplementary Figure 7: ROC curve reflecting the prediction accuracy of the logistic model developed by the relative abundances of Bacteroidetes and Firmicutes.

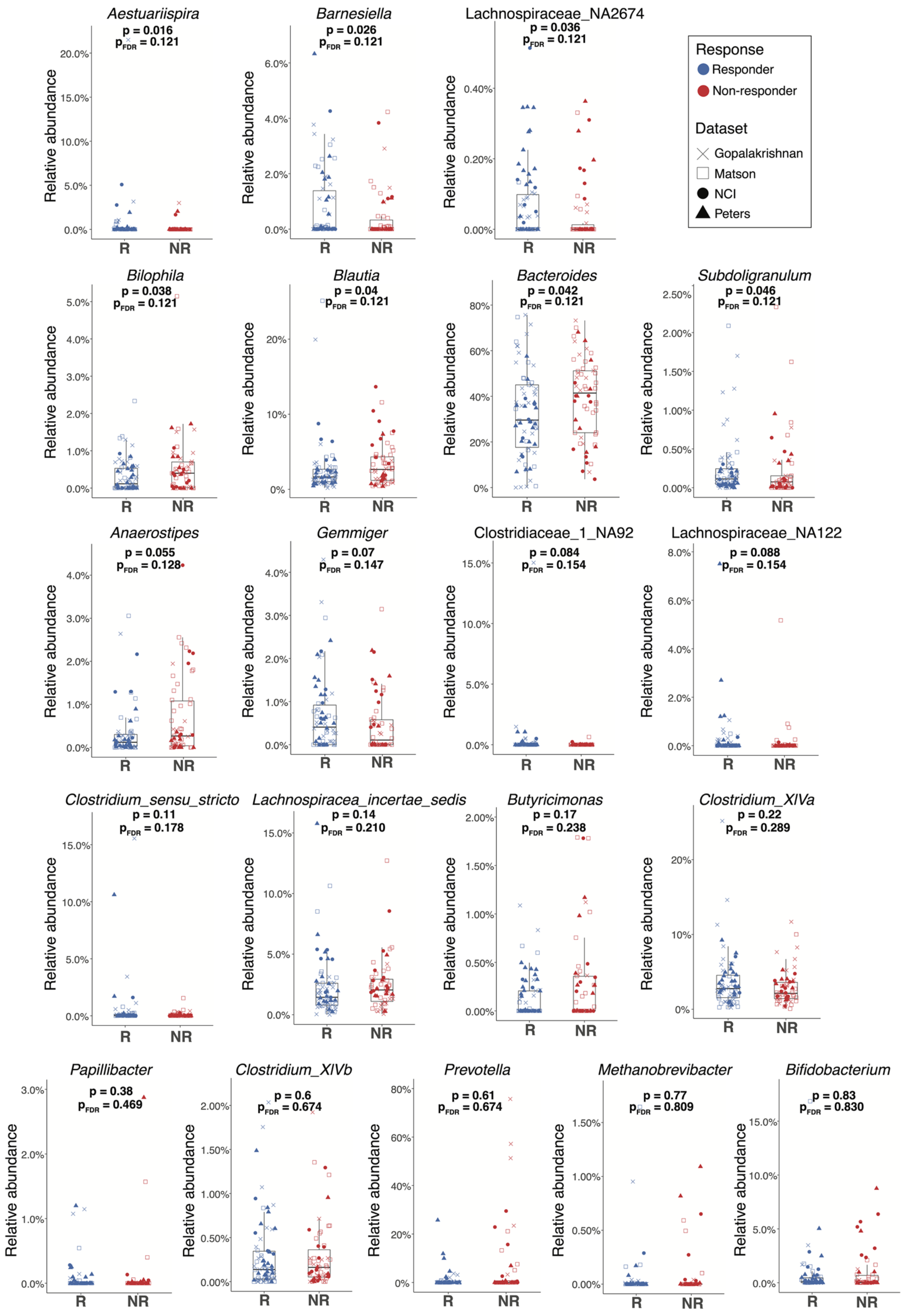

Supplementary Figure 8: Box plots showing the relative abundances of *selbal*-selected genera from the gut microbiome of all samples grouped by response type (Wilcoxon rank-sum test, unadjusted and FDR-corrected *p* values). Boxes represent the first and third quartiles. Upper and lower whiskers extend from the box hinge to the largest/smallest value no further than 1.5*IQR.

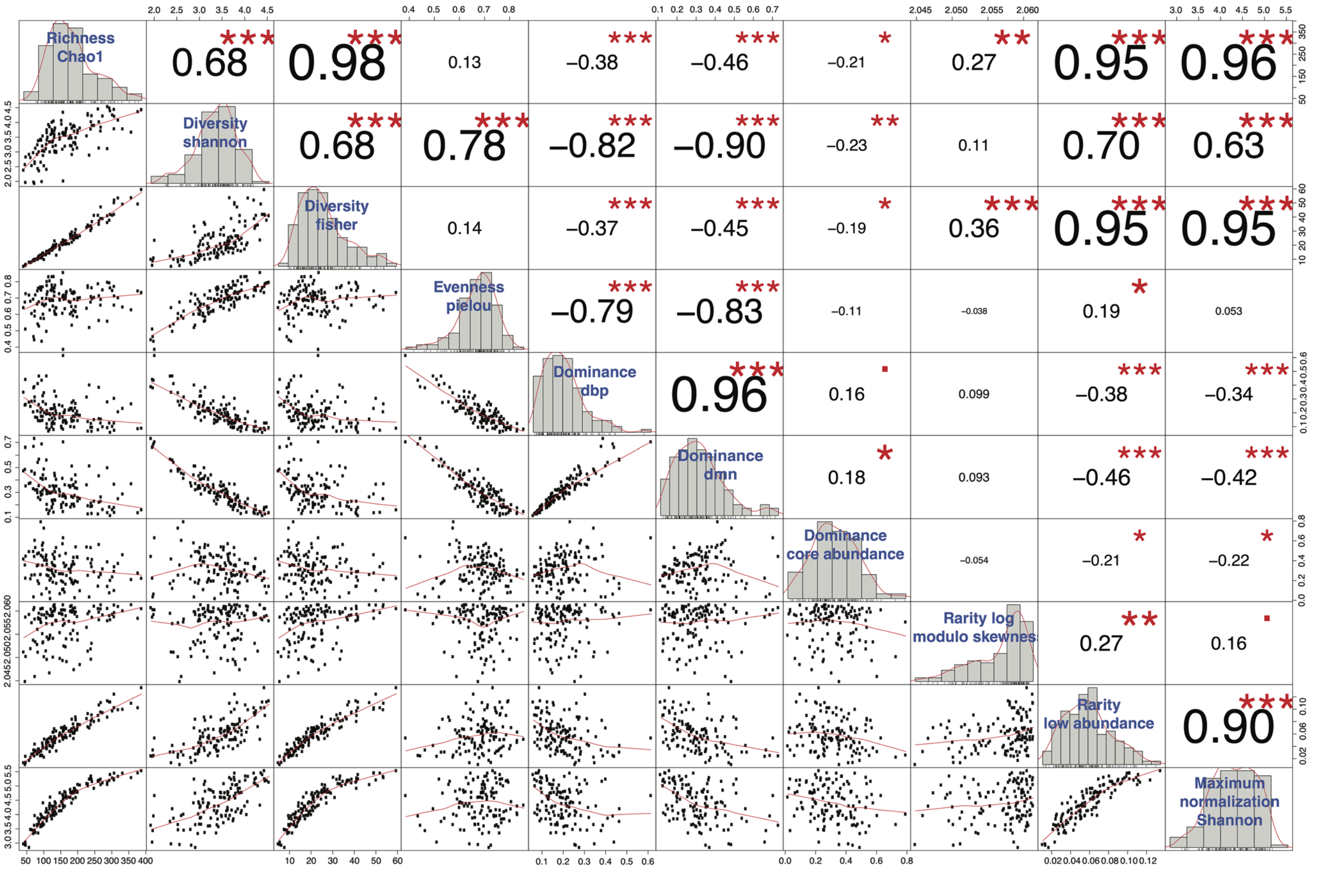

Supplementary Figure 9: Spearman correlation matrix among the diversity indexes used for statistical modeling. Distributions of each index are shown as bivariate scatter plots with a fitted line on the bottom half of the matrix. The coefficients of the spearman correlation plus the significance levels are shown on the top half of the matrix. For the symbols associated with significance levels, "***", "**", "*", "." and " ", represent *p* values of 0.001, 0.01, 0.05, 0.1 and 1, respectively.

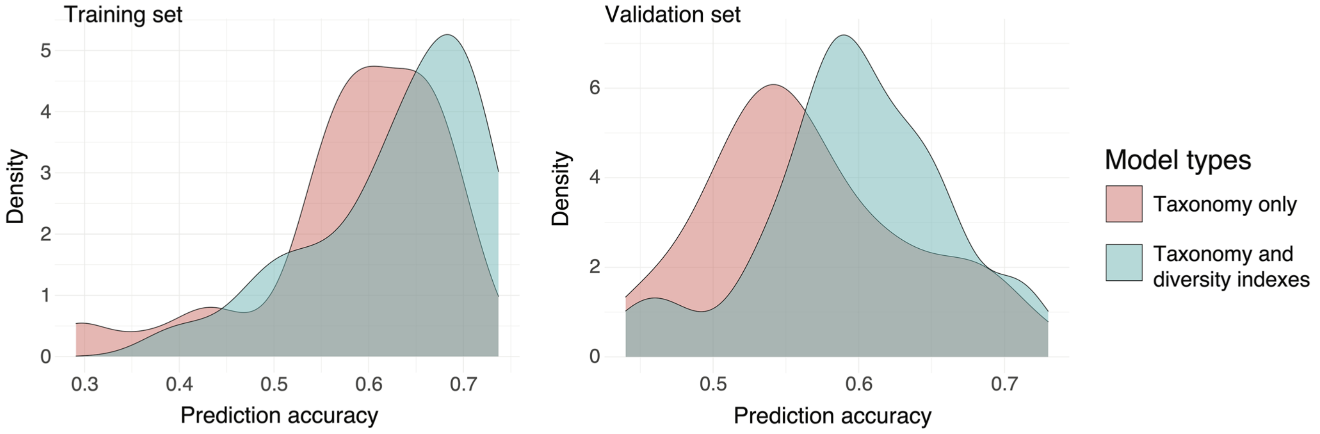

Supplementary Figure 10: Density plot showing the overall prediction accuracies of statistical models developed using taxonomy signals only or taxonomy signals plus diversity indexes. Left: Prediction accuracies from the training set in cross-validation. Right: Prediction accuracies when applying the models to the validation set.

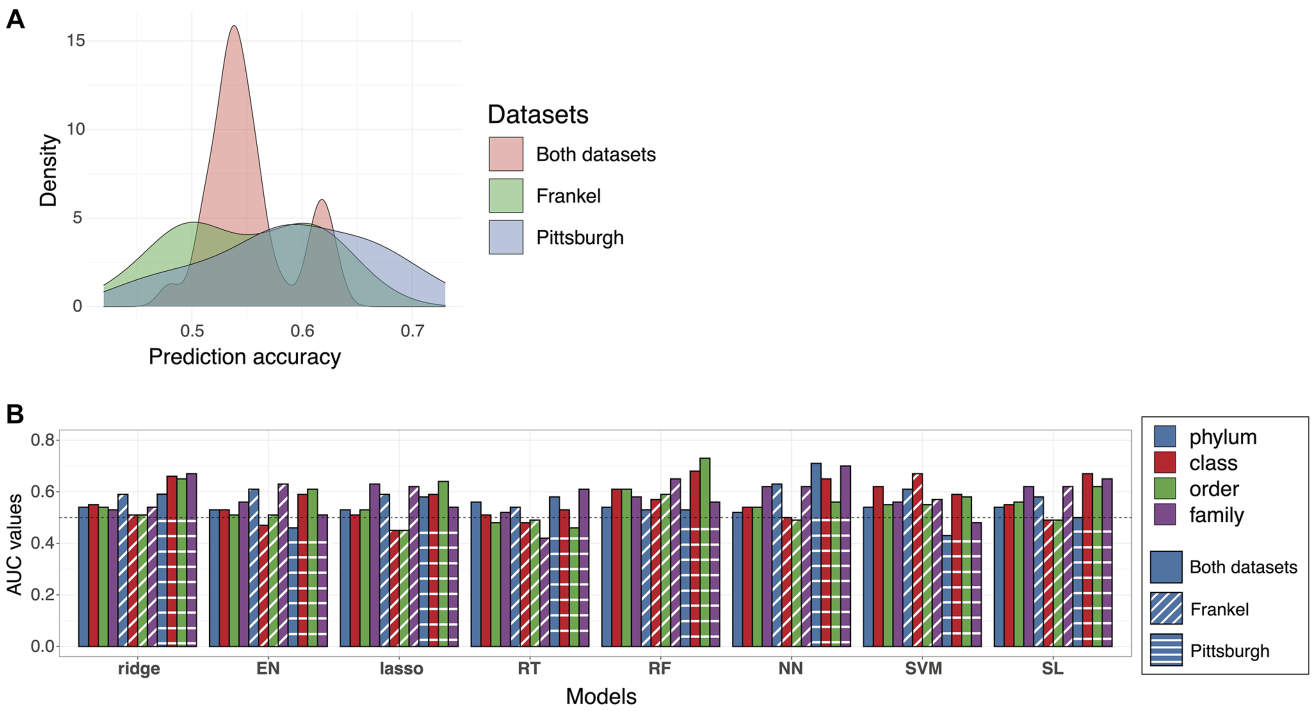

Supplementary Figure 11: Performance of the models when tested with shotgun metagenomic sequencing datasets. (**A**) Density plot showing the overall prediction accuracies of statistical models when working with each dataset or combined datasets. (**B**) Bar plot of the AUC values with colors representing different taxa levels and stripes representing the datasets.

Supplementary Table 1: Demographic profile of patients in NCI cohort

		All patients (N = 16)	%	Responders (N = 6)	%	Non Responders (N = 10)	%
**Sex**	Female	3	19%	1	17%	2	20%
	Male	12	75%	5	83%	7	70%
	Other	1	6%	0	0%	1	10%
**Age at sampling (years)**	<50	4	25%	1	17%	3	30%
	50–70	10	63%	5	83%	5	50%
	71+	2	13%	0	0%	2	20%
**Cancer Type**	Prostate	4	25%	2	33%	2	20%
	Sarcomatoid Renal Cell	1	6%	0	0%	1	10%
	Adenoid Cystic	2	13%	0	0%	2	20%
	Bronchiopulmonary Carcinoid	1	6%	1	17%	0	0%
	Pancreatic	3	19%	1	17%	2	20%
	Colon	1	6%	0	0%	1	10%
	Chordoma	1	6%	0	0%	1	10%
	Anal	2	13%	2	33%	0	0%
	Bladder	1	6%	0	0%	1	10%
**Treatment Type**	Anti-PDL1	5	31%	2	33%	3	30%
	Anti-PDL1 TRAP	10	63%	4	67%	6	60%
	Cabo+Nivo	1	6%	0	0%	1	10%
**Prior antibiotic use**	None	10	63%	4	67%	6	60%
	≤30 days prior to sampling	4	25%	0	0%	4	40%
	>30 days prior to sampling	2	13%	2	33%	0	0%

Abbreviations: PDL1: programmed death ligand 1; Cabo+Nivo: cabozantinib + nivolumab.

Supplementary Tables 2–14 and STORMS Checklist: Available at https://github.com/skinmicrobiome/gut-microbiome-ML

Supplementary Table 2: The taxonomic profile of gut bacterial community members from NCI cohort. See Supplementary Table 2.

Supplementary Table 3: Sequence of OTU signals at the species level for un-assigned ASVs from NCI cohort. See Supplementary Table 3.

Supplementary Table 4: Sequence of OTU signals at the genus level for un-assigned ASVs from NCI cohort. See Supplementary Table 4.

Supplementary Table 5: Statistically significant taxa signals with > 2-fold change between responders and non-responders. See Supplementary Table 5.

Supplementary Table 6: Summary of the datasets used for the microbiome analysis and statistical modeling. See Supplementary Table 6.

Supplementary Table 7: The taxonomic profile of gut bacterial community members from combined datasets. See Supplementary Table 7.

Supplementary Table 8: Sequence of OTU signals at the species level for un-assigned ASVs from combined datasets. See Supplementary Table 8.

Supplementary Table 9: Sequence of OTU signals at the genus level for un-assigned ASVs from combined dataset. See Supplementary Table 9.

Supplementary Table 10: All major taxa signals and alpha diversity indexes used for statistical modeling. See Supplementary Table 10.

Supplementary Table 11: AUC estimates (standard errors) of statistical models developed with training set in cross-validation. See Supplementary Table 11.

Supplementary Table 12: AUC estimates based on the validation set of 40 patients (standard error ranged between 0.08 and 0.10 in all cases). See Supplementary Table 12.

Supplementary Table 13: All major taxa signals from shotgun metagenomic sequencing datasets used for model validation. See Supplementary Table 13.

Supplementary Table 14: AUC estimates based on shotgun metagenomic sequencing datasets (standard error ranged between 0.06 and 0.08 for the combined dataset, between 0.08 and 0.10 for the Frankel et al., and between 0.10 and 0.13 for the McCulloch et al., dataset). See Supplementary Table 14.
